# Prognostic Value of SYNTAX Score II in Patients with Acute Coronary Syndromes Referred for Invasive Management: A Subanalysis from the SPUM and COMFORTABLE AMI Cohorts

**DOI:** 10.1155/2018/9762176

**Published:** 2018-09-25

**Authors:** Slayman Obeid, Antonio H. Frangieh, Lorenz Räber, Nooraldaem Yousif, Thomas Gilhofer, Kyohei Yamaji, Milosz Jaguszewski, Soheila Aghlmandi, James Adams, Yannik Bockhorn, Christian Templin, Barbara E. Stähli, Peter Jüni, Nicolas Rodondi, François Mach, Marco Roffi, Stephan Windecker, Willibald Maier, Fabian Nietlispach, Christian M. Matter, Roland Klingenberg, Thomas F. Lüscher

**Affiliations:** ^1^University Heart Center, Department of Cardiology, Zurich, Switzerland; ^2^Cardiovascular Center, Department of Cardiology, University Hospital Bern, Bern, Switzerland; ^3^Institute of Social and Preventive Medicine and Clinical Trials Unit, Department of Clinical Research, University of Bern, Bern, Switzerland; ^4^Department of General Internal Medicine, University Hospital Bern, Bern, Switzerland; ^5^Institute of Primary Health Care (BIHAM), University of Bern, Bern, Switzerland; ^6^Cardiovascular Center, Department of Cardiology, University Hospital Geneva, Geneva, Switzerland; ^7^Royal Brompton & Harefield Hospital Trust, Imperial College London, London, UK

## Abstract

**Aims:**

To assess the incremental prognostic value of SYNTAX score II (SxSII) as compared to anatomical SYNTAX Score (SxS) and GRACE risk score in patients with acute coronary syndromes who underwent percutaneous coronary intervention.

**Methods and results:**

SxSII and SxS were determined in 734 ACS patients. Patients were enrolled in the prospective Special Program University Medicine ACS and the COMFORTABLE AMI cohorts and later on stratified according to tertiles of SxSII (SxSII_Low_ ≤21.5 (*n*=245), SxSII_Mid_ 21.5–30.6 (*n*=245), and SxSII_High_ ≥30.6 (*n*=244). The primary endpoint of adjudicated all-cause mortality and secondary endpoints of MACE (cardiac death, repeat revascularization, and myocardial infarction) and MACCE (all-cause mortality, cerebrovascular events, MI, and repeat revascularization) were determined at 1-year follow-up. SxSII provided incremental predictive information for risk stratification when compared to SxS and GRACE risk score (AUC 0.804, 95% CI 0.77–0.84, *p* < 0.001 versus 0.67, 95% CI 0.63–0.72, *p*=0.007 versus 0.69, 95% CI 0.6–0.8, *p*=0.002), respectively. In a multivariable Cox regression analysis, we found that unlike SxS (adjusted HR 1.013, 95% CI (0.96–1.07), *p*=0.654), SxSII was significantly associated with all-cause mortality (HR = 1.095, 95% CI (1.06–1.11), *p* < 0.001). This was also true for the prediction of both secondary outcomes MACE (*n*=60) and MACCE (*n*=70) with an adjusted HR = 1.055, 95% CI (1.03–1.08), *p* < 0.001, and HR = 1.065, 95% CI (1.04–1.09), *p* < 0.001.

**Conclusion:**

In patients with ACS who underwent PCI, SxSII is an independent predictor of mortality during 1-year follow-up. SxSII shows superiority in discriminating risk compared to conventional SxS and GRACE for all-cause mortality.

## 1. Introduction

The anatomical SYNTAX (Synergy between percutaneous coronary intervention with taxus and cardiac surgery) score (SxS) is an angiographic scoring system for assessing the complexity of coronary artery disease (CAD) [1] advocated for decision making in the latest ESC/EACTS guidelines on myocardial revascularization [2]. Originally, the SxS was introduced to predict clinical outcomes in stable patients with 3-vessel and/or left main disease undergoing percutaneous coronary intervention (PCI) or coronary artery bypass grafting (CABG), respectively, based on data from the SYNTAX trial [3, 4]. Later on, the SxS was applied to a variety of patient populations with diverse clinical presentations including those with acute coronary syndromes (ACS) undergoing primary PCI [5, 6].

However, subanalyses of the SYNTAX trial and results from different studies have implied that the purely anatomy-based risk stratification of the SxS score made it prone to misclassification of patient's true risk, particularly for all-cause mortality and cardiac death in patients with stable CAD or ACS treated by PCI [7, 8]. The addition of clinical variables was a promising step in improving risk stratification by reclassifying patients into more accurate risk categories. Therefore, in order to account for the variability of clinical parameters affecting long-term outcomes and hence better classification of patients' risk, the SYNTAX score II (SxSII) was developed by complementing SxS with 7 prognostic variables including age, creatinine clearance, left ventricular ejection fraction (LVEF), presence of unprotected left main coronary artery (ULMCA) disease, peripheral vascular disease (PVD), female gender, and chronic obstructive pulmonary disease (COPD) [9]. So far, the SxSII was validated in patients with left main and multivessel disease showing more accurate patient stratification than SxS [10, 11, 12].

The aim of the present study was to assess the predictive performance of SxSII in patients presenting with ACS undergoing PCI and to compare it to the previously validated SxS and the commonly used Global Registry of Acute Coronary Events (GRACE) risk score [13].

## 2. Methods

### 2.1. Study Population

The prospective multicenter Special Program University Medicine—Acute Coronary Syndromes and Inflammation (SPUM-ACS, ClinicalTrials.gov number, NCT01000701) enrolled consecutive patients who were referred for coronary angiography with a diagnosis of ACS to one of the participating Swiss university hospitals (Zurich, Bern, Lausanne, and Geneva) between December 2009 and October 2012 [14, 15]. Inclusion criteria comprised patients of both genders, aged ≥18 years, presenting within 5 days (preferably within 72 hours) after pain onset with a main diagnosis of STEMI, NSTEMI, or unstable angina. Enrolled patients had symptoms compatible with angina pectoris (chest pain and dyspnea) and fulfilled at least one of the following criteria: (a) ECG changes, such as persistent ST-segment elevation or depression, T-inversion or dynamic ECG changes, or new left bundle branch block (LBBB); (b) evidence of positive (predominantly conventional) troponin by local laboratory reference values (with a rise and/or fall in serial troponin levels); and (c) known coronary artery disease specified by its status after MI, coronary artery bypass graft (CABG), or PCI or newly documented ≥50% stenosis of an epicardial coronary artery during the initial catheterization. Exclusion criteria for the SxSII study comprised prior CABG, referral to either CABG or medical management after completion of the coronary angiogram, severe physical disability, and inability to comprehend study or less than 1 year of life expectancy for noncardiac reasons. Within this consortium, a centralized electronic database was implemented providing comprehensive information on all patients comprising both clinical and coronary anatomy parameters. A telephone follow-up was performed at 30 days and at 1 year, a clinical visit. Adverse events occurring within 365 days after the index ACS event were adjudicated by an independent adjudication committee consisting of 3 experienced cardiologists (Lukas Kappenberger, MD, Lausanne; Tiziano Moccetti, MD Lugano; and Mathias E. Pfisterer, MD, Basel). An additional 3-year follow-up to assess all-cause mortality was ascertained by telephone for the SxSII study, and only patients recruited in Bern and Zurich were analyzed in the SxSII study. The study was approved by the local ethical committees, and all patients gave written informed consent in compliance with the Declaration of Helsinki.

The COMFORTABLE AMI trial included patients aged 18 years or older who had a history of chest pain of more than a 10 min duration and associated ST-segment elevation of >1 mm in ≥2 contiguous leads, new left bundle branch block, or true posterior MI, who underwent primary percutaneous coronary intervention (PCI) within 24 h of symptom onset. In addition, there was angiographic presence of at least one acute infarct-related artery (IRA) with one or multiple coronary artery lesions in a native coronary artery with a diameter between 2.25 and 4.0 mm, which could be treated with one or multiple stents. Exclusion criteria included the use of vitamin K antagonists, mechanical complications of myocardial infarction, acute myocardial infarction secondary to stent thrombosis (ST), planned surgery within 6 months of PCI unless dual antiplatelet therapy could be maintained throughout the perisurgical period, and noncardiac comorbid conditions with life expectancy <1 year. Further study details are described in detail elsewhere [16].

Angiography was digitally recorded and analyzed in a central core laboratory. The MI SxS score was assessed by experienced analysts using the web-based program http://www.syntaxscore.com as previously described. Angiographic documentation of patients included in the COMFORTABLE AMI trial was scored as described previously.

### 2.2. Clinical Outcomes

All-cause mortality included cardiac, vascular, and noncardiovascular causes of death. Cerebrovascular events comprised stroke (any, ischemic, hemorrhagic, and unclear etiology) or transient ischemic attack (TIA); repeat revascularization included any repeat coronary revascularization (target and nontarget vessel). Clinically indicated repeat revascularization included any clinically driven repeat coronary revascularization (target and nontarget vessel) [17]. Myocardial infarction was defined based on the universal definition including periprocedural MI in patients with UA [18].

The primary endpoint of our study was adjudicated all-cause mortality at 1-year follow-up. The secondary endpoints were adjudicated major adverse cardiovascular events (MACE) defined as the composite of cardiac death, clinically indicated revascularization, or MI at 1 year and adjudicated major adverse cardiovascular and cerebrovascular events (MACCE) defined as the composite of all-cause mortality, cerebrovascular events, any repeat revascularization, or myocardial infarction (MI) at 1-year, respectively.

### 2.3. Anatomical SYNTAX Score, SYNTAX Score II, and GRACE Risk Score Calculation

Experienced cardiologists blinded to clinical outcomes assessed the SYNTAX score for each angiogram. The interobserver and intraobserver variabilities of the SxS scoring team were previously reported as moderate (kappa statistic 0.56) and substantial (kappa statistic 0.70, respectively). The intraclass correlation coefficient for calculated SXS in SPUM cohort for absolute agreement was 0.886 (*p* < 0.001), 95% CI (0.835, 0.919). Before accessing any lesions, all those with ≥50% diameter stenosis in vessels ≥1.5 mm in diameter were scored using the SxS algorithm [1]. The SxSII was then calculated using the PCI SYNTAX score II (http://www.syntaxscore.com) calculator based on the previously published nomogram [9], with scores assigned for the presence and magnitude of each predictor specific for PCI population [9, 11]. The GRACE risk score to calculate long-term mortality comprised age, heart rate, systolic blood pressure, initial serum creatinine, history of congestive heart failure, history of myocardial infarction, elevated cardiac markers (conventional troponins as per local laboratories), ST-segment depression, and no in-hospital PCI [13]. The long-term GRACE risk score was calculated using a program written in STATA, and we used the standard scoring of GRACE as mentioned in the reference publications. (http://www.wikidoc.org/index.php/The_GRACE_risk_score). It was retrospectively calculated for all patients included in our study and was compared to SxSII and SxS as a continuous variable by ROC curve analysis and multivariable Cox regression model.

### 2.4. Statistical Analysis

Continuous variables are expressed as mean ± SD or medians with interquartile ranges (IQR) and were compared using one-way ANOVA, Student's *t*-test, Kruskal–Wallis test, or Mann–Whitney test as appropriate. Categorical data are presented as frequency (percentages) and were compared using the Fisher exact or the chi-square test. Cumulative incidences were calculated using Kaplan–Meier curves.

For our analysis, we stratified patients according to tertiles of SxSII [9] (≤21.5, 21.5–30.6, ≥30.6) and SXS (≤12, 12–22, ≥22). The score ranges are referred to as SxSII_Low_, SxSII_Mid_, and SxSII_High_ and SxS_Low_, SxS_Mid_, and SxS_High_, respectively. We constructed multivariable Cox proportional hazard models including variables that had a significant association with a *p* value of <0.05 in univariable analysis. We further added SxS and SxSII separately into the model due to their collinearity. Other variables showed no multicollinearity that exceeding the acceptable threshold of VIF ≥3 or tolerance ≤0.2. From the full model, we selected variables to minimize Akaike's information criterion by backward stepwise methods. Calibration was then determined by the Hosmer–Lemeshow goodness-of-fit test. To visualize the effect of SxSII on clinical outcomes and confirm its linearity, we constructed an alternative model using penalized splines. Analyses were performed with SPSS version 21.0 software (SPSS Inc., Chicago, Ill).

ROC curves were constructed to assess the ability of the SxSII, SxS, and GRACE risk score to predict events at 1-year follow-up. Patients who were lost to follow-up at 1 year were excluded from the analysis. Areas under curves were compared using the DeLong method [19] provided by MedCalc for Windows, version 14.10.2 (MedCalc Software, Acacialaan, Belgium). Category-free net reclassification improvement (NRI) and integrated discrimination improvement (IDI) were both calculated using the “survIDINRI,” R package (R-version 3.3.2), through comparing proportional hazards models, whereas category-based NRI was done using MATLAB version R2015b, all as described by Pencina et al. [20]. A probability value of <0.05 was considered significant, and all tests were two-tailed.

## 3. Results

### 3.1. Baseline and Angiographic Characteristics for Tertiles of SYNTAX Score II

SxS and SxSII could be calculated for all 734 patients with a complete one-year follow-up. The mean anatomical SYNTAX score was 17.56 ± 9.3 with a median of 16 with an interquartile range (IQR) of 13. Patients were categorized into SxS tertiles (SxS_Low_, *n*=251, SxS_Mid_, *n*=244, and SxS_High_, *n*=239). The mean SYNTAX score II was 27.7 ± 10.3 with a median of 25.4. The number of patients stratified according to tertiles of SxSII_Low_, SxSII_Mid_, and SxSII_High_, was 245, 245, and 244, respectively. The tertiles of SxSII and its individual components including the anatomical SYNTAX score are listed in Table 1 as they are compared to tertiles of the conventional SxS.

Compared with patients in the lower tertiles, patients in SxSII_High_ had a higher rate of adverse cardiovascular history and risk factors and fasting glucose, along with hemodynamic instability on admission. The baseline characteristics and risk factors of patients according to SxSII tertiles are listed in Table 2. Angiographic characteristics showed significant differences with a higher rate of MVD with left anterior descending artery (LAD) involvement (74.2%) in the SxSII_High_ tertile compared with patients in the lower tertiles (Table 3).

### 3.2. Clinical Outcomes Stratified by Tertiles of Anatomical SYNTAX and SYNTAX Score II

In order for better assessment of the impact SxSII has on mortality, we expressed this relationship in terms of penalized splines curves (hazard ratio-based curves) obtained through a Cox proportional hazard regression model (Supplementary Figure 1). As the relationship was curve-linear, our choice of breaking the score into tertiles was justified.

Patients within the SxSII_High_ tertile had a significantly higher incidence of all-cause mortality, MACE, and MACCE compared with patients in lower tertiles (Table 4). There was also a higher rate of clinically driven revascularization, in SxSII_High_ (8.6% *p*=0.002) with a trend for excess cerebrovascular events, *p*=0.134. One-year outcomes across the tertiles of SxS are reported in Supplementary Table 1.

**Figure 1:**
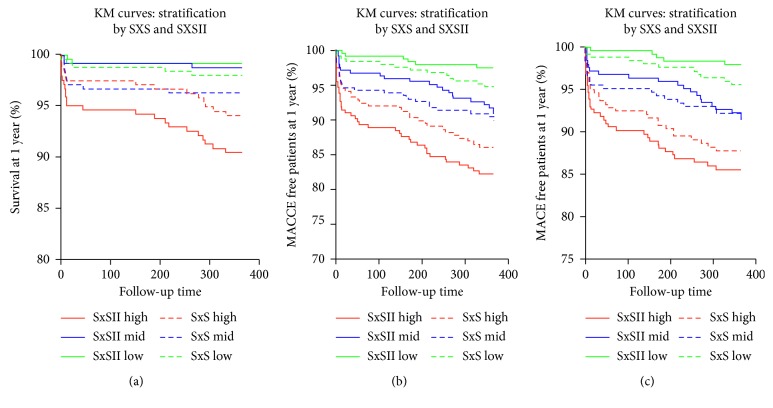
Kaplan–Meier survival curves at 1-year follow-up for freedom from all-cause mortality, MACCE, and MACE.

Furthermore, Kaplan–Meier curves were plotted to asses all studied outcomes across tertiles of both SxS and SxSII as shown in Figure 1. All-cause mortality (9.4% versus 1.2% versus 0.8%), MACCE (17.6% versus 8.6% versus 2.4%), and MACE (14.3% versus 8.2% versus 2%) occurred at a significantly higher rate among patients in SxSII_High_ compared to SxSII_Mid_ and SxSII_Low_, respectively (*p* (log rank) <0.001). Conversely, the anatomical SYNTAX score does not provide consistent risk stratification, specifically when addressing the primary endpoint of all-cause mortality at 1 year.

### 3.3. A Comparison between SxSII, SxS, and GRACE Score

**Figure 2:**
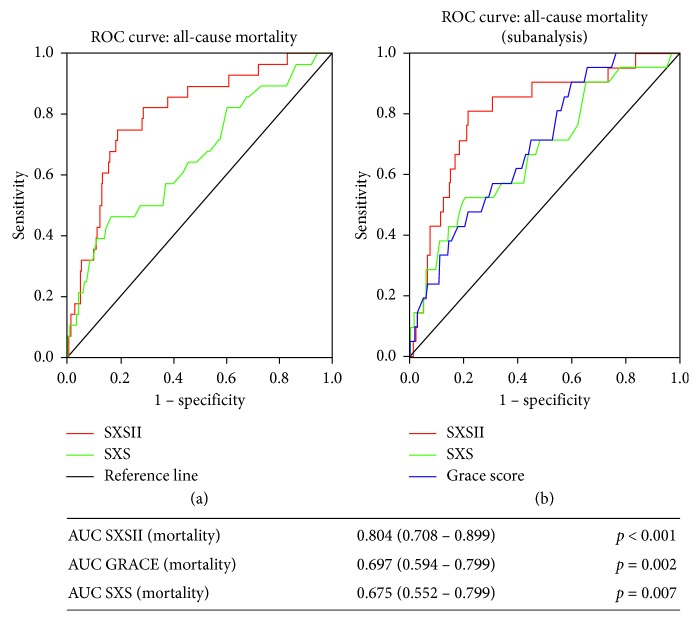
ROC curves. (a) Receiver-operating characteristic (ROC) curves for SYNTAX score II and anatomical SYNTAX in predicting 1-year all-cause mortality (entire population of 734 patients). SxSII (red line) significantly improves prediction over both scores. AUC = area under the curve; CI = confidence intervals. (b) Receiver-operating characteristic (ROC) curves for SYNTAX score II, anatomical SYNTAX, and GRACE risk score in predicting 1-year all-cause mortality (subanalysis of 500 patients).

ROC curves showed an improved area under the curve (AUC) when comparing SxSII with SxS regarding all-cause mortality at 1 year (0.803 (0.773–0.831) versus 0.658 (0.622–0.692)) (Figure 2). This improvement was statistically significant at an AUC difference of 0.145 (95% CI 0.049–0.246, *p*=0.0045). Compared with the GRACE risk score (calculated in 500 patients), SxSII showed a persistently higher prognostic accuracy for all-cause mortality (Figure 2). Conversely, prognostic accuracy for MACE during 1-year follow-up (AUC (0.657 (0.621–0.691) versus 0.684 (0.649–0.718), *p*_Difference_=0.475) was not different between SxS and SxSII (Supplementary Figure 2).

**Figure 3:**
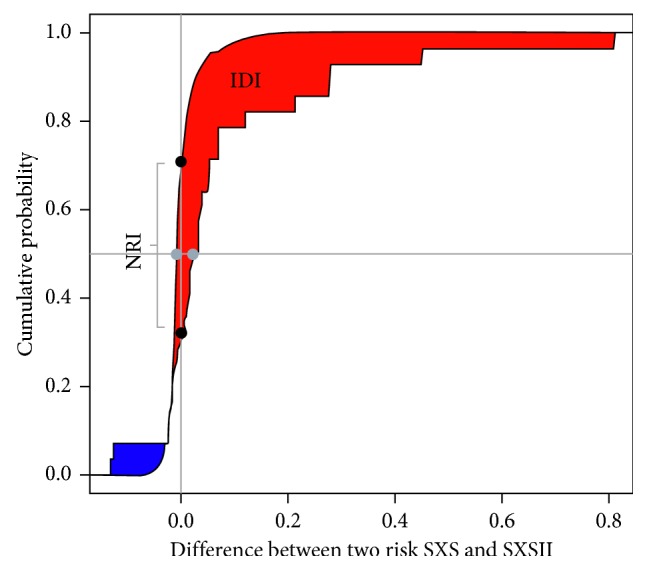
Category-free IDI and NRI. The red shaded area shows a significant integrated discrimination improvement (IDI), whereas the difference between the two vertical dots represents the improvement in net reclassification by SXSII over SxS.

Additionally, we sought to authenticate the benefit in risk assessment, that we have seen so far by SxSII over SXS, through performing a category-free and category-based net reclassification improvement (NRI) along with integrated discrimination improvement (IDI) for the outcome of all-cause mortality at 1 year. We found a significant categorical net reclassification improvement of 0.344 (*p*=0.004) with Z score of 2.714 for all-cause mortality, primarily driven by a significant net gain of 0.321 (*p*=0.006) in patients who had the event and a trend (*p*=0.39) in patients without the event (Table 5). More importantly, both estimates of category-free net reclassification improvement (NRI) and integrated discrimination improvement (IDI) were significant (*p* < 0.001) at 0.380 95% CI (0.191–0.554) and 0.08 95% CI (0.035–0.278), respectively. The red shaded area and its extent show a substantial added value of the SxSII model over SxS (Figure 3).

### 3.4. Results from Multivariable Analysis

For model construction, variables which were found to correlate significantly with the studied outcomes were plotted (Supplementary Figures 3 and 4) as part of hazard assessment in a univariable Cox regression analysis. The anatomical SYNTAX score, age, LVEF, and GFR were predictive of the primary outcome; however, they correlated significantly with SxSII while at the same time showing multicollinearity exceeding the acceptable threshold of VIF<3. Hence, two separate models for SxSII and SxS were constructed, showing the SxSII to be a significant predictor of all-cause mortality (adjusted HR 1.095 95% CI (1.05–1.13), *p* < 0.001) along with the presence of left main disease, fasting glucose, and resuscitation status (Table 6). On the other hand, the anatomical SYNTAX score got short of significance for the prediction of our primary outcome (adjusted HR 1.013 95% CI (0.956–1.073), *p*=0.656). The complete list of predictors in multivariable analysis for the outcomes of MACE and MACCE is shown in Supplementary Table 2.

Additional analysis regarding the impact of categorizing patients within tertiles of SxSII showed that a classification into SxSII_High_ independently predicted all-cause mortality (HR 12.48, 95% CI (2.61−59.01], *p*=0.002), MACCE (HR 7.310, 95% CI (2.90−18.3), *p* < 0.001), and MACE (HR 6.64, 95% CI (2.42–18.18), *p* < 0.001) as compared to the reference category SxSII_Low_.

Further evaluation of the value of the SxSII over the established GRACE risk score, which can be assessed without information on coronary anatomy, was conducted through an additional multivariable Cox regression model to predict 1-year all-cause mortality. The results showed that SxSII unlike GRACE score remained to be significantly predictive of mortality (adjusted HR 1.061 95% CI (1.01–1.11), *p*=0.014) (Supplementary Table 3).

## 4. Discussion

To the best of our knowledge, this is the first study to evaluate and compare the novel SxSII score in patients with ACS undergoing PCI with the currently available SxS and GRACE risk scores. The following key findings were obtained:

- SxSII independently predicts all-cause mortality, MACE, and MACCE during 1-year follow-up.
- SxSII provides superior discrimination of risk for all-cause mortality and MACCE than the conventional SxS and GRACE score, respectively.

In the current era of interventional cardiology, the role of the multidisciplinary *HeartTeam* in choosing the optimal available means of revascularization is emphasized by current ESC guidelines [2] advocating the use of the anatomical SYNTAX score as a fundamental tool to assist in the decision making for surgical versus percutaneous coronary revascularization in stable coronary artery disease patients with left main or multivessel disease [2]. However, risk assessment and the prediction of long-term outcomes in patients presenting with ACS undergoing PCI is still suboptimal with a wide range of old and newly emerging risk scores [10].

The SxS has been extensively studied for a variety of clinical outcomes in different patient populations including all-comers [21, 22] as well as patients with NSTEMI [23, 24] or STEMI [25, 26]. The recently developed SxSII has been complemented with clinically significant prognostic variables, known to be independent predictors of mortality at 4 years in patients with stable CAD enrolled in the SYNTAX trial [9, 25, 27]. In that patient population, this was translated into better discrimination of risk for long-term mortality for SxSII when compared to SxS [9]. The main findings from our study support the superiority of SxSII also in patients with ACS undergoing PCI who are at particular risk as it was found to be an independent predictor of all-cause mortality during 1-year follow-up. In contrast, the anatomical SYNTAX score was not an independent predictor for this endpoint, in line with previous studies [22, 28, 29]. The discrepant findings between studies evaluating the anatomical SYNTAX score to predict adverse clinical outcome (particularly all-cause mortality) are likely attributable to the heterogeneous patient population analyzed (inclusion or exclusion of patients with STEMI and/or cardiogenic shock). Indeed, the latter patients tend to have a worse outcome which is not predicted by anatomical complexity alone. Furthermore, different cutoffs used in statistical models to calculate SxS may explain the observed differences.

To further substantiate the significant improvement in discrimination or risk for all-cause mortality identified for SxSII, we compared this score to the clinically based, ESC-advocated risk stratification in patients with NSTEMI [2], the GRACE risk score [13]. Despite the fact that patients with cardiogenic shock with high GRACE risk scores were included in our cohort, the SxSII showed greater discrimination of risk for all-cause mortality during 1-year follow-up. The GRACE risk score lacks some clinical variables which are important predictors of mortality, such as LVEF, as well as classifiers of complex coronary anatomy [30–33], which may explain the reduced prognostic accuracy to predict all-cause mortality when compared with the SxSII.

In our study, SxSII also showed good prognostic accuracy for the secondary endpoint of MACCE. Conversely, SxSII provided no incremental risk stratification for MACE compared with SxS. This could be explained by the fact that MACE, which were primarily attributable to MI and clinically indicated revascularization in a population with complex coronary anatomy (i.e., a mean SxS of 17.56 ± 9.3, 44% MVD and 63.5% LAD involvement), could be well predicted by pure anatomical complexity [34–36] as assessed by SxS. Indeed, in our multivariable analysis, multivessel disease was a predictor of MACE and MACCE when assessed with both models of SxSII. However, it fell short of significance for all-cause mortality along with the anatomical which is concordant with previously published studies [6].

Importantly, SxSII enabled reclassification of risk for all-cause mortality compared with SxS. This was highly significant for 1-year follow-up with a persistent net gain of approximately 33%. Upon exploring the differences in complexity of the coronary anatomy between tertiles of SxSII and SxS, patients would still classify in the middle category of the original anatomical SxS, although the anatomical SYNTAX score in the SxSII_High_ group was higher as compared to that in SxSII_Mid_ and SxSII_Low_. Therefore, the use of SxSII for risk stratification in patients with ACS undergoing PCI identifies a distinctive group of patients who despite having moderately complex coronary artery disease are still at a significantly higher risk of both cardiovascular and noncardiovascular death during follow-up. These are patients with multiple risk factors and comorbidities who may benefit from intensive secondary prevention, particularly from some of the most recent lipid-lowering drugs [14, 37‐ 39] and risk factor modification with closer follow-up intervals and may derive an advantage of full revascularization when multivessel disease is present.

Surprisingly enough, diabetes was not a predictor of clinical outcomes, which could be explained by the fact that end-organ damage secondary to diabetes—as reflected by scores reflecting coronary anatomy and calcification—rather than metabolic parameters better reflect risk in this patient population [9, 13, 40]. Interestingly, abnormal fasting glucose levels during hospitalization for the index event were predictive of all-cause mortality irrespective of the diabetes status of the patients. Indeed, in line with previous studies, fasting glucose was also an independent variable capable of predicting all-cause mortality in this population [41–44]. The pathophysiological mechanisms are well described elsewhere [45–47], including but not limited to the effects on the collateral circulation, infarct size, reperfusion, sympathetic activation with elevated catecholamine levels leading to hepatic glucose release and platelet aggregation.

### 4.1. Study Limitations

One limitation of our study is the fact that due to the low number of patients undergoing CABG or medical therapy in this ACS population, risk by SxSII in the primary PCI group could not be compared to these different treatment modalities. Furthermore, since the SxSII was developed using prognostic variables in a population where STEMI patients were excluded, there may be additional parameters that could further improve risk stratification of adverse clinical outcome.

## 5. Conclusion

In the present study, we demonstrate a clinically relevant superiority of the novel SYNTAX score II when compared to the anatomical SYNTAX and commonly used GRACE risk score, in risk stratification of patients with ACS undergoing PCI. Pending validation in other cohorts, our data suggest that the use of the SYNTAX score II opens a new door for improvement in decision making and management of patients with ACS.

## Figures and Tables

**Table 1:** Baseline characteristics and risk factors.

Variables	SxSII_Low_ ≤21.5	SxSII_Mid_ 21.5–30.6	SxSII_High_ ≥30.6	*p* value
	*N*=245	*N*=245	*N*=244	

Age^*∗*^	52.3 ± 7.4	60.5 ± 9.5	71.7 ± 10.2	N/A
Gender (male)^*∗*^	239 (97.6)	205 (83.7)	146 (59.8)	N/A
ACS				0.001
STEMI	167 (68.2)	181 (73.9)	189 (77.5)	
NSTE-ACS	65 (26.5)	63 (25.7.4)	53 (21.7)	
Unstable angina	13 (5.3)	1 (0.4)	2 (0.8)	
Hypertension	100 (43.1)	122 (51.7)	155 (68)	<0.001
History of dyslipidemia	143 (61.9)	140 (59.6)	138 (60.8)	0.878
Diabetes mellitus	28 (12.1)	41 (17.4)	39 (17.1)	0.207
History of smoking	207 (84.5)	188 (76.7)	142 (59.2)	<0.001
History of CAD	72 (31.9)	57 (24.2)	38 (17.5)	0.002
Prior MI	11 (4.7)	20 (8.5)	25 (11)	0.049
Prior PCI	19 (8.2)	25 (10.5)	28 (12.3)	0.347
History of PVD^*∗*^	0 (0)	1 (0.4)	23 (9.4)	N/A
History of COPD^*∗*^	3 (1.2)	6 (2.4)	15 (6.1)	N/A
Killip score ≥2	21 (8.5)	31 (12.7)	46 (18.9)	0.003
Prehospital resuscitation	16 (6.5)	8 (3.3)	10 (4.1)	0.226
Vasopressors	5 (2)	4 (1.6)	15 (6.1)	0.007
IABP	3 (1.2)	9 (3.7)	33 (13.5)	<0.001
BMI (kg/m^2^)	27.7 ± 3.9	27.5 ± 4.2	25.6 ± 3.6	<0.001
HsTnT (ug/l)	0.14 (0.04/0.51)	0.18 (0.04/0.67)	0.24 (0.08/0.73)	0.008
CK-MB (U/l)	24.3 (11/57)	28.5 (12/68)	34 (14/66)	0.104
NT-proBNP (ng/l)	137 (47/311)	256 (95/701)	586 (195/1960)	<0.001
eGFR (ml/min/1.73 m^2^)^*∗*^	127.8 ± 30.8	106.9 ± 33.6	69.5 ± 29.4	N/A
Fasting glucose (mmol/l)	6.8 ± 2.2	7.4 ± 2.9	7.8 ± 3.4	0.001
LVEF (%)^*∗*^	55.7 ± 7.4	50.7 ± 10.2	45.9 ± 12.4	N/A

Depicted are counts, *n* incidence (%) or mean ± SD or median (25/75) percentile. ^*∗*^Variables are included in the score; *p* value reported as N/A. CAD, coronary artery disease; MI, myocardial infarction; PCI, percutaneous coronary intervention; PVD, peripheral vascular disease; COPD, chronic obstructive pulmonary disease; LVEF, left ventricular ejection fraction.

**Table 2:** Components of SYNTAX score II.

Variables	SxSII_Low_ ≤21.5	SxSII_Mid_ 23–32	SxSII_High_ ≥30.6	*p* value
	*N*=245	*N*=245	*N*=244	

Age	52.3 ± 7.4	60.5 ± 9.5	71.7 ± 10.2	<0.001
Gender (male)	239 (97.6)	205 (83.7)	146 (59.8)	<0.001
PVD	0 (0)	1 (0.4)	23 (9.4)	<0.001
COPD	3 (1.2)	6 (2.4)	15 (6.1)	0.004
eGFR (ml/min/1.73 m^2^)	127.8 ± 30.8	106.9 ± 33.6	69.5 ± 29.4	<0.001
LVEF (%)	55.7 ± 7.4	50.7 ± 10.2	45.9 ± 12.4	<0.001
LM	2 (0.8)	2 (0.8)	10 (4.1)	0.009
Anatomical SYNTAX score (SxS)	12.6 ± 6.9	17.6 ± 7.8	22.4 ± 10.2	<0.001

Variables	SxS_Low_ ≤12	SxS_Mid_ 12-22	SxS_High_ ≥22	*p* value
	*N*=251	*N*=244	*N*=239	

Anatomical SYNTAX score (SxS)	7.9 ± 2.8	16.9 ± 2.91	28.5 ± 6.4	<0.001

PVD, peripheral vascular disease; COPD, chronic obstructive pulmonary disease; LVEF, left ventricular ejection fraction; GFR, glomerular filtration rate; LM, left main disease.

**Table 3:** Angiographic characteristics and medications.

Variables	SxSII_Low_ <23	SxSII_Mid_ 23–32	SxSII_High_ ≥23	*p* value
	*N*=176	*N*=164	*N*=160	

*Vessel involvement*				
LM^*∗*^	2 (0.8)	2 (0.8)	10 (4.1)	N/A
LAD	121 (49.4)	157 (64.1)	181 (74.2)	<0.001
LCx	72 (29.4)	83 (34)	84 (34.4)	0.419
RCA	125 (51)	126(51.6)	134 (54.9)	0.654

*MVD# of vessels*	86 (35.1)	106 (43.3)	130 (53.3)	<0.001
2 vessels	72(29.4)	71(29)	83(34)	
3 vessels	14(5.7)	35(14.3)	47(19.3)	

*ACC/AHA lesion classification*	0.413			0.413
Type A lesion	15 (9.6)	18 (11.7)	10 (7.0)	
Type B1 lesion	73 (46.8)	56 (36.4)	60 (42.3)	
Type B2 lesion	41 (26.3)	45 (29.2)	37 (26.1)	
Type C lesion	27(17.3)	35 (22.7)	35 (24.6)	

*Restenotic lesion*	7 (2.9)	6 (2.4)	5 (2.1)	0.876
*Bifurcation lesion*	20 (8.3)	28 (11.5)	27 (11.2)	0.441
*Thrombus Procedural details*	132 (54.3)	120(49)	107(44.2)	0.083
Length of 1st stent	25.4 ± 5	20.2 ± 5	25.4 ± 5	
Diameter of 1st stent	3.03 ± 0.68	2.9 ± 0.41	2.81 ± 0.22	
Length of 2nd stent	19.85 ± 6.83	23.6 ± 6.12	18.2 ± 5.6	
Diameter of 2nd stent	3.1 ± 0.28	3 ± 0.68	2.6 ± 0.13	
Length of 3rd stent	15.9 ± 7.02	12.4 ± 2.8	16.2 ± 9	
Diameter of 3rd stent	3.17 ± 0.31	2.95 ± 0.77	2.65 ± 0.48	

*TIMI flow*				0.207
TIMI 0	107 (44.4)	134 (54.9)	128 (53.1)	
TIMI I	20 (8.3)	15 (6.1)	10 (4.1)	
TIMI II	38 (15.8)	33 (13.5)	36 (14.9)	
TIMI III	76 (31.5)	62 (25.4)	67 (27.8)	

*Medications on admission*				
ASA	213 (86.9)	205 (83.7)	159 (65.7)	<0.001
Clopidogrel	6 (2.5)	7 (2.9)	14 (5.8)	0.125
Prasugrel/ticagrelor	1 (0.6)	0 (0)	0 (0)	1
Statin	34 (14)	48 (19.6)	63 (26.1)	0.004
Beta blocker	31(12.7)	43 (17.6)	68 (28.3)	<0.001

*Procedural antiplatelet Tx*				
Clopidogrel	119 (69.2)	120 (73.2)	117 (73.6)	0.451
Prasugrel	63 (35.8)	46 (28.0)	26 (16.4)	<0.001

Depicted are counts, *n* incidence (%) or mean ± SD.^*∗*^variables are included in the score; *p* value reported as N/A. LAD, left anterior descending artery; RCA, right coronary artery; LCX, circumflex artery; TIMI, thrombolysis in myocardial infarction; AHA, American Heart Association.

**Table 4:** Clinical outcomes at 1 year stratified by tertiles of SYNTAX score II.

Outcomes at one year	SxSII_Low_≤21.5	SxSII_Mid_ 21.5–30.6	SxSII_High_ ≥30.6	*p* value	*N*=734
	*N*=245	*N*=245	*N*=244		

All-cause mortality	2 (0.8)	3 (1.2)	23 (9.4)	<0.001	28 (3.8)
Cardiovascular death	1 (0.4)	3 (1.2)	17 (7.0)	<0.001	21 (2.8)
Noncardiovascular death	1 (0.4)	0 (0.0)	6 (2.4)	0.007	7 (0.9)
Cerebrovascular event	0 (0.0)	1 (0.4)	3 (1.2)	0.134	4 (0.5)
Myocardial infarction	1 (0.4)	9 (3.7)	10 (4.1)	0.011	20 (2.7)
Clinically driven revascularization	4 (1.6)	16 (6.5)	21 (8.6)	0.002	41 (5.6)
Target vessel revascularization	2 (1.7)	13(5.3)	13 (5.3)	0.011	28 (3.8)
Any revascularization	4 (1.6)	18(7.3)	21 (8.6)	0.002	43 (5.8)
Restenotic lesion revascularisation	3 (1.7%)	2 (1.2%)	9 (1.8%)	0.03	14 (1.9)
Any stent thrombosis	2 (1.1%)	0 (0%)	3 (1.9%)	0.59	5 (0.6)
Early thrombosis	1 (0.6%)	0 (0%)	2 (1.3%)	0.50	3 (0.4)
Late thrombosis	1 (0.6%)	0 (0%)	1 (0.6%)	0.77	2 (0.2)
MACE	5 (2.0)	20 (8.2)	35 (14.3)	<0.001	60 (7.4)
MACCE	6 (2.4)	21 (8.6)	43 (17.6)	<0.001	70(9.5)

MACE, major adverse cardiac events; MACCE, major adverse cardiac and cerebrovascular events.

**Table 5:** Category-based NRI by SxSII.

		Tertiles of SxSII			Number of patients
		Low	Mid	High	
Patients with an event					
Tertiles of SxS	Low	1	1	3	5
					17.90%
	Mid	1	2	6	9
					32.10%
	High	0	0	14	14
					50%
All-cause mortality		2	3	23	28
		7.10%	10.70%	21.10%	100.00%

Patients without an event					
Tertiles of SxS	Low	134	67	45	246
					34.80%
	Mid	79	97	59	235
					33.30%
	High	30	78	117	225
					31.90%

All-cause mortality		243	242	221	709
		34.40%	34.30%	31.10%	100.00%

Number of patients with events moving 1 scale up by SxSII = 10, number of patients with events moving 1 scale down by SxSII = 1, number of patients without events moving 1 scale down by SxSII = 187, and number of patients without events moving 1 scale up by SxSII = 171. Category-based NRI = 0.344, *z* = 2.833, *p*=0.004.

**Table 6:** Multivariable predictors of all-cause mortality at one year.

HR (95% CI)		*p* value
Variables in SxSII model		All-cause mortality
SxSII	1.095 (1.05–1.13)	<0.001
LM disease	4.825 (1.40–16.59)	0.0125
Fasting glucose	1.081 (1.01–1.15)	0.0268
Resuscitation	11.48 (4.53–29.06)	<0.001
Gender (male)	0.369 (0.126–1.08)	0.0685
H&L test: X^2^:3.156,df:8,p:0.9		

Variables in SXS model		All-cause mortality
Age	1.106 (1.05–1.16)	<0.0001
GFR	1.011 (0.99–1.02)	0.14
LVEF	0.937 (0.90–0.97)	<0.001
LM disease	3.491 (0.93–12.9)	0.06
Resuscitation	19.7 (6.97–55.6)	<0.0001
Fasting glucose	1.13 (1.05–1.23)	0.0015
SXS	1.013 (0.956–1.073)	0.656

## Data Availability

The data used to support the findings of this study are available from the corresponding author upon request.

## Abbreviations

ACS: Acute coronary syndromes
AUC: Area under the curve
CABG: Coronary artery bypass graft
CI: Confidence intervals
ECG: Electrocardiogram
ESC: European Society of Cardiology
GRACE: Global Registry of Acute Coronary Events
LBBB: Left bundle branch block
MI: Myocardial infarction
NSTEMI: Non-ST-elevation myocardial infarction
PCI: Percutaneous coronary intervention
ROC: Receiver-operating characteristics.

## Supplementary Materials

(Supplementary Materials) Supplementary Figure 1: spline curve representation of different cutoffs of SxSII score and their respective hazard for the outcome of all-cause mortality. Supplementary Figure 2: ROC curves. Supplementary Figures 3 and 4: univariable predictors of studied outcomes. Supplementary Table 1: clinical outcomes at 1 year stratified by tertiles of anatomical SYNTAX score. Supplementary Table 2: multivariable predictors of MACE and MACCE at one year. Supplementary Table 3: GRACE versus SxSII score in prediction of 1-year all-cause mortality.

## References

[1] Sianos G., Morel M. A., Kappetein A. P. (2005). The SYNTAX Score: an angiographic tool grading the complexity of coronary artery disease. *EuroIntervention: Journal of EuroPCR in Collaboration with the Working Group on Interventional Cardiology of the European Society of Cardiology*.

[2] Authors/Task Force members, Windecker S., Kolh P. (2014). ESC/EACTS Guidelines on myocardial revascularization: the task force on myocardial revascularization of the European Society of Cardiology (ESC) and the european association for cardio-thoracic surgery (EACTS) developed with the special contribution of the European association of percutaneous cardiovascular interventions (EAPCI). *European Heart Journal*.

[3] Serruys P. W., Onuma Y., Garg S. (2009). Assessment of the SYNTAX score in the Syntax study. *EuroIntervention*.

[4] Serruys P. W., Morice M. C., Kappetein A. P. (2009). Percutaneous coronary intervention versus coronary-artery bypass grafting for severe coronary artery disease. *New England Journal of Medicine*.

[5] Capodanno D., Di Salvo M. E., Cincotta G., Miano M., Tamburino C., Tamburino C. (2009). Usefulness of the SYNTAX score for predicting clinical outcome after percutaneous coronary intervention of unprotected left main coronary artery disease. *Circulation Cardiovascular Interventions*.

[6] Caixeta A., Genereux P., Palmerini T. (2014). Prognostic utility of the SYNTAX score in patients with single versus multivessel disease undergoing percutaneous coronary intervention (from the acute catheterization and urgent intervention triage strategy [ACUITY] trial). *American Journal of Cardiology*.

[7] Serruys P. W., Farooq V., Vranckx P. (2012). A global risk approach to identify patients with left main or 3-vessel disease who could safely and efficaciously be treated with percutaneous coronary intervention: the SYNTAX Trial at 3 years. *JACC Cardiovascular Interventions*.

[8] Keelan P. C., Johnston J. M., Koru-Sengul T. (2003). Comparison of in-hospital and one- year outcomes in patients with left ventricular ejection fractions <or = 40%, 41% to 49%, and >or = 50% having percutaneous coronary revascularization. *American Journal of Cardiology*.

[9] Farooq V., van Klaveren D., Steyerberg E. W. (2013). Anatomical and clinical characteristics to guide decision making between coronary artery bypass surgery and percutaneous coronary intervention for individual patients: development and validation of SYNTAX score II. *The Lancet*.

[10] Farooq V., Head S. J., Kappetein A. P., Serruys P. W. (2014). Widening clinical applications of the SYNTAX Score. *Heart*.

[11] Campos C. M., van Klaveren D., Iqbal J. (2014). Predictive performance of SYNTAX score II in patients with left main and multivessel coronary artery disease-analysis of CREDO-Kyoto registry. *Circulation Journal*.

[12] Xu B., Genereux P., Yang Y. (2014). Validation and comparison of the long-term prognostic capability of the SYNTAX score-II among 1,528 consecutive patients who underwent left main percutaneous coronary intervention. *JACC: Cardiovascular interventions*.

[13] Eagle K. A., Lim M. J., Dabbous O. H. (2004). A validated prediction model for all forms of acute coronary syndrome: estimating the risk of 6-month postdischarge death in an international registry. *JAMA*.

[14] Gencer B., Montecucco F., Nanchen D. (2016). Prognostic value of PCSK9 levels in patients with acute coronary syndromes. *European Heart Journal*.

[15] Klingenberg R., Heg D., Raber L. (2015). Safety profile of prasugrel and clopidogrel in patients with acute coronary syndromes in Switzerland. *Heart*.

[16] Magro M., Raber L., Heg D. (2014). The MI SYNTAX score for risk stratification in patients undergoing primary percutaneous coronary intervention for treatment of acute myocardial infarction: a substudy of the COMFORTABLE AMI trial. *International Journal of Cardiology*.

[17] Cutlip D. E., Windecker S., Mehran R. (2007). Clinical end points in coronary stent trials: a case for standardized definitions. *Circulation*.

[18] Thygesen K., Alpert J. S., White H. D. (2007). Joint ESC/ACCF/AHA/WHF expert consensus document: universal definition of myocardial infarction. *Journal of the American College of Cardiology*.

[19] DeLong E. R., DeLong D. M., Clarke-Pearson D. L. (1988). Comparing the areas under two or more correlated receiver operating characteristic curves: a nonparametric approach. *Biometrics*.

[20] Pencina M. J., D’Agostino R. B., D’Agostino R. B., Vasan R. S. (2008). Evaluating the added predictive ability of a new marker: from area under the ROC curve to reclassification and beyond. *Statistics in Medicine*.

[21] Wykrzykowska J. J., Garg S., Girasis C. (2010). Value of the SYNTAX score for risk assessment in the all-comers population of the randomized multicenter LEADERS (Limus Eluted from A Durable versus ERodable Stent coating) trial. *Journal of the American College of Cardiology*.

[22] Girasis C., Garg S., Raber L. (2011). SYNTAX score and Clinical SYNTAX score as predictors of very long-term clinical outcomes in patients undergoing percutaneous coronary interventions: a substudy of SIRolimus-eluting stent compared with pacliTAXel- eluting stent for coronary revascularization (SIRTAX) trial. *European Heart Journal*.

[23] Palmerini T., Genereux P., Caixeta A. (2011). Prognostic value of the SYNTAX score in patients with acute coronary syndromes undergoing percutaneous coronary intervention: analysis from the ACUITY (Acute Catheterization and Urgent Intervention Triage StrategY) trial. *Journal of the American College of Cardiology*.

[24] Farooq V., Vergouwe Y., Genereux P. (2013). Prediction of 1-year mortality in patients with acute coronary syndromes undergoing percutaneous coronary intervention: validation of the logistic clinical SYNTAX (Synergy Between Percutaneous Coronary Interventions With Taxus and Cardiac Surgery) score. *JACC cardiovascular Interventions*.

[25] Garg S., Sarno G., Serruys P. W. (2011). Prediction of 1-year clinical outcomes using the SYNTAX score in patients with acute ST-segment elevation myocardial infarction undergoing primary percutaneous coronary intervention: a substudy of the STRATEGY (Single High-Dose Bolus Tirofiban and Sirolimus-Eluting Stent Versus Abciximab and Bare-Metal Stent in Acute Myocardial Infarction) and MULTISTRATEGY (Multicenter Evaluation of Single High-Dose Bolus Tirofiban Versus Abciximab With Sirolimus-Eluting Stent or Bare-Metal Stent in Acute Myocardial Infarction Study) trials. *JACC Cardiovascular Interventions*.

[26] Akgun T., Oduncu V., Bitigen A. (2014). Baseline SYNTAX Score and long-term outcome in patients with ST-segment elevation myocardial infarction undergoing primary percutaneous coronary intervention. *Clinical and Applied Thrombosis*.

[27] Farooq V., Serruys P. W., Bourantas C. (2012). Incidence and multivariable correlates of long-term mortality in patients treated with surgical or percutaneous revascularization in the synergy between percutaneous coronary intervention with taxus and cardiac surgery (SYNTAX) trial. *European Heart Journal*.

[28] Brener S. J., Prasad A. J., Abdula R., Sacchi T. J (2011). Relationship between the angiographically derived SYNTAX score and outcomes in high-risk patients undergoing percutaneous coronary intervention. *Journal of Invasive Cardiology*.

[29] Chakravarty T., Buch M. H., Naik H. (2011). Predictive accuracy of SYNTAX score for predicting long-term outcomes of unprotected left main coronary artery revascularization. *American Journal of Cardiology*.

[30] Lev E. I., Kornowski R., Vaknin-Assa H. (2008). Comparison of the predictive value of four different risk scores for outcomes of patients with ST-elevation acute myocardial infarction undergoing primary percutaneous coronary intervention. *American Journal of Cardiology*.

[31] Taniwaki M., Stefanini G. G., Silber S. (2014). 4-year clinical outcomes and predictors of repeat revascularization in patients treated with new-generation drug- eluting stents: a report from the RESOLUTE All- Comers trial (A Randomized Comparison of a Zotarolimus-Eluting Stent With an Everolimus-Eluting Stent for Percutaneous Coronary Intervention). *Journal of the American College of Cardiology*.

[32] Navarese E. P., Kolodziejczak M., Kereiakes D. J., Tantry U. S., O’Connor C., Gurbel P. A. (2016). Proprotein convertase subtilisin/kexin Type 9 monoclonal antibodies for acute coronary syndrome: a narrative review. *Annals of Internal Medicine*.

[33] Sabatine M. S., Giugliano R. P., Wiviott S. D. (2015). Efficacy and safety of evolocumab in reducing lipids and cardiovascular events. *New England Journal of Medicine*.

[34] Muhlestein J. B., Anderson J. L., Horne B. D. (2003). Effect of fasting glucose levels on mortality rate in patients with and without diabetes mellitus and coronary artery disease undergoing percutaneous coronary intervention. *American Heart Journal*.

[35] Giraldez R. R., Clare R. M., Lopes R. D. (2013). Prevalence and clinical outcomes of undiagnosed diabetes mellitus and prediabetes among patients with high-risk non-ST- segment elevation acute coronary syndrome. *American Heart Journal*.

[36] Savonitto S., Morici N., Cavallini C. (2014). One-year mortality in elderly adults with non-ST-elevation acute coronary syndrome: effect of diabetic status and admission hyperglycemia. *Journal of the American Geriatrics Society*.

[37] Deedwania P., Kosiborod M., Barrett E. (2008). Hyperglycemia and acute coronary syndrome: a scientific statement from the American Heart Association Diabetes Committee of the Council on Nutrition, Physical Activity, and Metabolism. *Circulation*.

[38] Morice M. C., Serruys P. W., Kappetein A. P. (2010). Outcomes in patients with de novo left main disease treated with either percutaneous coronary intervention using paclitaxel– eluting stents or coronary artery bypass graft treatment in the Synergy Between Percutaneous Coronary Intervention with TAXUS and Cardiac Surgery (SYNTAX) trial. *Circulation*.

[39] Kappetein A. P., Feldman T. E., Mack M. J. (2011). Comparison of coronary bypass surgery with drug-eluting stenting for the treatment of left main and/or three-vessel disease: 3-year follow-up of the SYNTAX trial. *European Heart Journal*.

[40] Park S. J., Kim Y. H., Park D. W. (2011). Randomized trial of stents versus bypass surgery for left main coronary artery disease. *New England Journal of Medicine*.

[41] Capodanno D., Stone G. W., Morice M. C., Bass T. A., Tamburino C. (2011). Percutaneous coronary intervention versus coronary artery bypass graft surgery in left main coronary artery disease: a meta-analysis of randomized clinical data. *Journal of the American College of Cardiology*.

[42] Farooq V., Vergouwe Y., Raber L. (2012). Combined anatomical and clinical factors for the long-term risk stratification of patients undergoing percutaneous coronary intervention: the Logistic Clinical SYNTAX score. *European Heart Journal*.

[43] Garg S., Sarno G., Garcia-Garcia H. M. (2010). A new tool for the risk stratification of patients with complex coronary artery disease: the clinical SYNTAX score. *Circulation Cardiovascular Interventions*.

[44] Chen S. L., Chen J. P., Mintz G. (2010). Comparison between the NERS (new risk stratification) score and the SYNTAX (synergy between percutaneous coronary intervention with taxus and cardiac surgery) score in outcome prediction for unprotected left main stenting. *JACC Cardiovascular Interventions*.

[45] Farooq V., Brugaletta S., Serruys P. W. (2011). Contemporary and evolving risk scoring algorithms for percutaneous coronary intervention. *Heart*.

[46] Capodanno D., Miano M., Cincotta G. (2010). EuroSCORE refines the predictive ability of SYNTAX score in patients undergoing left main percutaneous coronary intervention. *American Heart Journal*.

[47] Feldman D. N., Gade C. L., Slotwiner A. J. (2006). Comparison of outcomes of percutaneous coronary interventions in patients of three age groups (<60, 60 to 80, and >80 years) (from the New York State Angioplasty Registry). *American Journal of Cardiology*.

