# Supplementary material for: Prognostic Value of SYNTAX Score II in Patients with Acute Coronary Syndromes Referred for Invasive Management: A Subanalysis from the SPUM and COMFORTABLE AMI Cohorts
Source: Cardiol Res Pract. 2018 Sep 25;2018:9762176. doi: 10.1155/2018/9762176 (PMC6176297; doi:10.1155/2018/9762176)
Supplement: (Supplementary Materials) — Supplementary Figure 1: spline curve representation of different cutoffs of SxSII score and their respective hazard for the outcome of all-cause mortality. Supplementary Figure 2: ROC curves. Supplementary Figures 3 and 4: univariable predictors of studied outcomes. Supplementary Table 1: clinical outcomes at 1 year stratified by tertiles of anatomical SYNTAX score. Supplementary Table 2: multivariable predictors of MACE and MACCE at one year. Supplementary Table 3: GRACE versus SxSII score in prediction of 1-year all-cause mortality. [file 9762176.f1.pdf]

Supplementary Figure 1. Spline curve representation of different cutoffs of SxSII score and their respective hazard for the outcome of all-cause mortality.

Above the median cutoff, (SxSII=25), there is a persistent increase in hazard for death at one year along with a significant improvement in the 95% confidence interval.

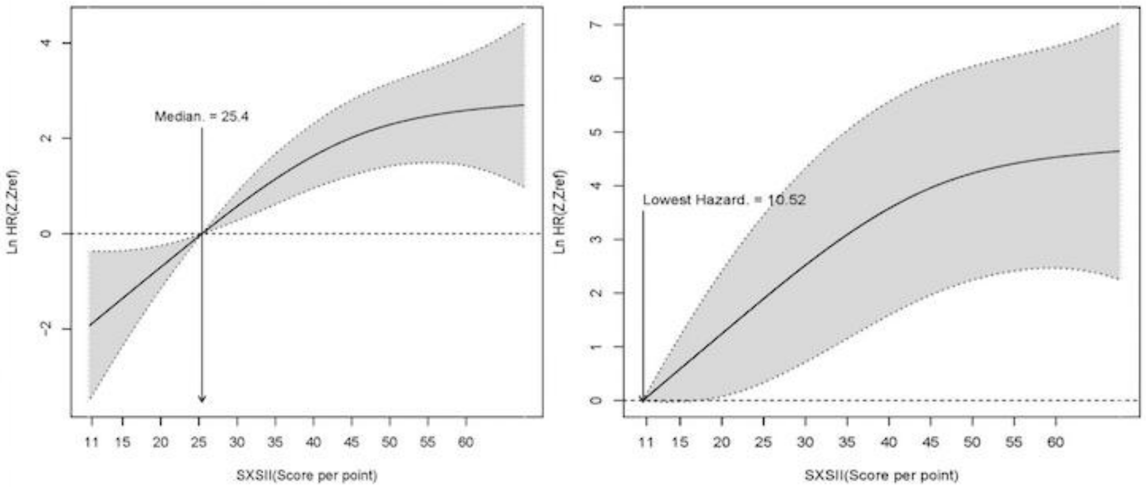

SXSII Score	Log Hazard	Lower 95% CI	Higher 95% CI
11	0.06320911	-0.006925241	0.1333435
15	0.58927967	-0.022237634	1.2007970
20	1.24445200	0.075598028	2.4133060
25	1.89463487	0.334068806	3.4552009
30	2.52056494	0.714365918	4.3267640
35	3.09028874	1.150103045	5.0304744
40	3.57660744	1.586886446	5.5663284
50	4.23265088	2.242573451	6.2227283
60	4.53298374	2.466540845	6.5994266

Supplementary Figure 2. ROC curves

Receiver-operating characteristic (ROC) curves for SYNTAX II, and anatomical SYNTAX in predicting 1-year MACE (Entire population of 734 patients). No significant difference between S_xSII (Red line) and SxS in prediction of 1-year MACE. AUC = area under curve; CI = confidence interval(s).

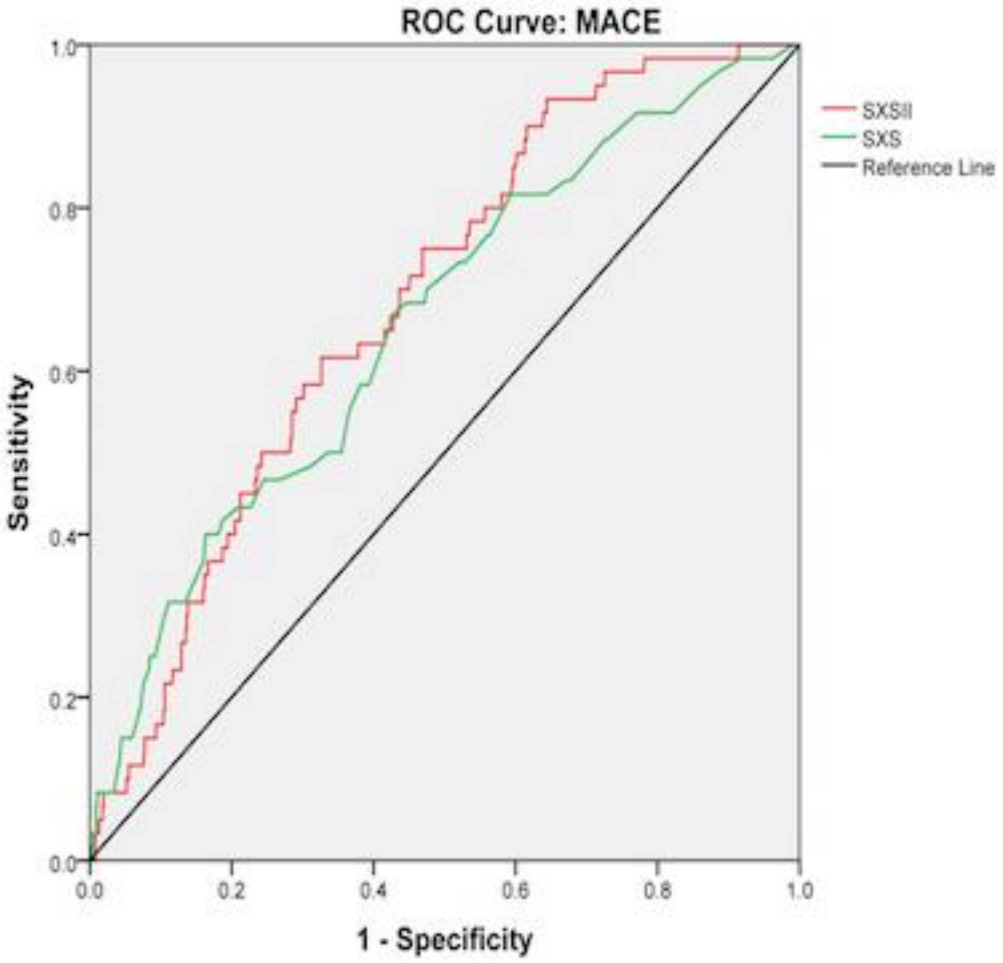

Supplementary Figures 3 and 4. Univaraible predictors of studied outcomes.

Shown here are different variables found to be significant in a univariaible analysis for the primary and secondary outcomes.

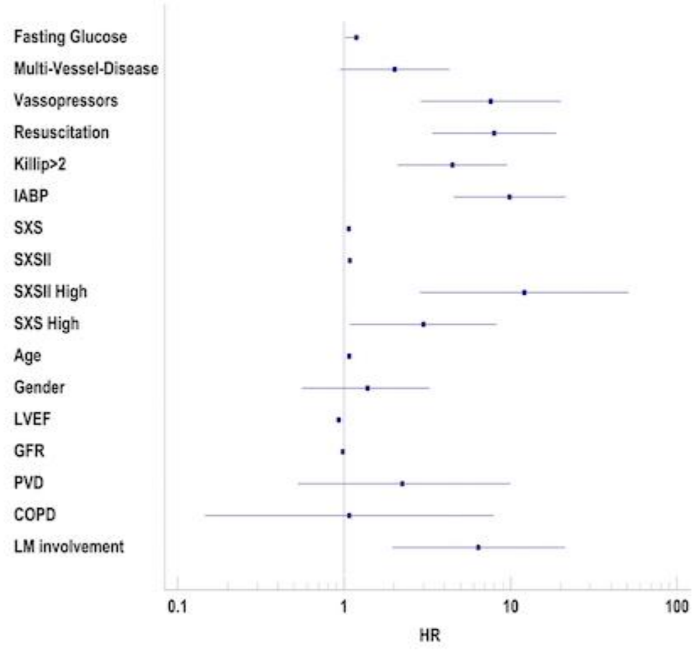

HR	95%CI	P-Value
1.168	1.101 – 1.240	<0.001
2.005	0.939 – 4.280	0.072
7.594	2.885 – 19.958	<0.001
7.952	3.379 – 18.713	<0.001
4.459	2.088 – 9.521	<0.001
9.877	4.557 – 21.407	<0.001
1.064	1.029 – 1.101	<0.001
1.085	1.056 – 1.115	<0.001
12.120	2.85 – 51.407	0.001
2.998	1.080 – 8.323	0.035
1.073	1.038 – 1.109	<0.001
1.383	0.588 – 3.254	0.457
0.930	0.899 – 0.964	<0.001
0.984	0.973 – 0.994	0.003
2.231	0.829 – 9.938	0.274
1.072	0.146 – 7.891	0.945
6.429	1.941 – 21.297	0.002

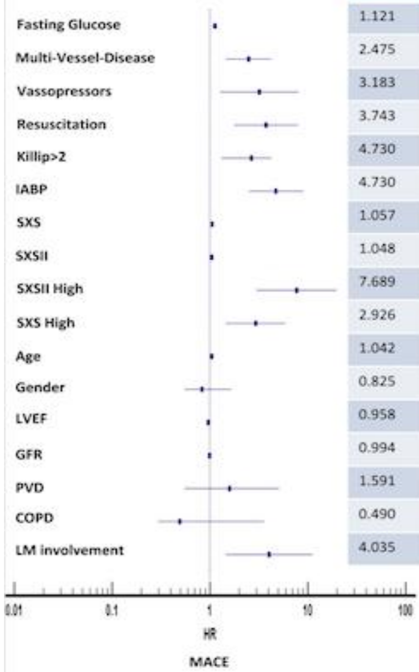

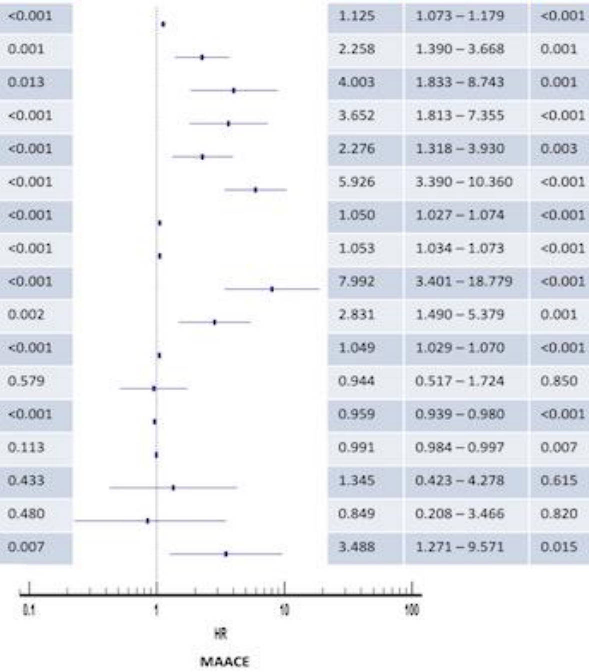

	MACE HR	MACE 95%CI	MACE P-Value	MAACE HR	MAACE 95%CI	MAACE P-Value
Fasting Glucose	1.121	1.064 – 1.180	<0.001	1.125	1.073 – 1.179	<0.001
Multi-Vessel-Disease	2.475	1.456 – 4.209	0.001	2.258	1.390 – 3.668	0.001
Vassopressors	3.183	1.274 – 7.954	0.013	4.003	1.833 – 8.743	0.001
Resuscitation	3.743	1.778 – 7.880	<0.001	3.652	1.813 – 7.355	<0.001
Killip>2	4.730	2.511 – 8.908	<0.001	2.276	1.318 – 3.930	0.003
IABP	4.730	2.511 – 8.908	<0.001	5.926	3.390 – 10.360	<0.001
SXS	1.057	1.033 – 1.083	<0.001	1.050	1.027 – 1.074	<0.001
SXSII	1.048	1.027 – 1.069	<0.001	1.053	1.034 – 1.073	<0.001
SXSII High	7.689	3.012 – 19.629	<0.001	7.992	3.401 – 18.779	<0.001
SXS High	2.926	1.462 – 5.858	0.002	2.831	1.490 – 5.379	0.001
Age	1.042	1.020 – 1.064	<0.001	1.049	1.029 – 1.070	<0.001
Gender	0.825	0.419 – 1.627	0.579	0.944	0.517 – 1.724	0.850
LVEF	0.958	0.936 – 0.981	<0.001	0.959	0.939 – 0.980	<0.001
GFR	0.994	0.988 – 1.001	0.113	0.991	0.984 – 0.997	0.007
PVD	1.591	0.498 – 5.079	0.433	1.345	0.423 – 4.278	0.615
COPD	0.490	0.068 – 3.538	0.480	0.849	0.208 – 3.466	0.820
LM involvement	4.035	1.463 – 11.130	0.007	3.488	1.271 – 9.571	0.015

Supplementary Table 1. Clinical Outcomes at 1-year stratified by tertiles of Anatomical SYNTAX Score

**Outcomes at one year**	**SxS $_{low}$ ≤12** N=251	**SxS $_{Mid}$ 12-22** N=244	**SxS $_{High}$ ≥22** N=239	**P-value**	**N=734**
All-cause Mortality	5 (2.0)	9 (3.7)	14 (5.9)	0.09	28 (3.8)
Cardiovascular Death	3 (1.2)	7 (2.9)	11 (4.6)	0.07	21 (2.8)
Non-Cardiovascular Death	2 (0.8)	2 (0.8)	3 (1.3)	1	7 (0.9)
Cerebrovascular Event	1 (0.4)	1 (0.4)	2 (0.8)	0.699	4 (0.5)
Myocardial Infarction	6(2.4)	2 (0.8)	12 (5.0)	0.017	20 (2.7)
Target Vessel Revascularization	8 (3.2)	8 (3.3)	12 (5.0)	0.498	28 (3.8)
Clinically Driven Revascularization	8 (3.2)	14 (5.7)	19 (7.9)	0.065	41 (5.6)
Any Revascularization	9(3.6)	15(6.1)	19 (7.9)	0.12	43 5.8)
MACE	11 (4.4)	20 (8.2)	29 (12.1)	0.007	60 (7.4)
MACCE	13 (5.2)	24 (9.8)	33 (13.8)	0.005	70 (9.5)

MACE, major adverse Cardiac events; MACCE, major adverse cardiac and cerebrovascular events

Supplementary Table 2. Multivariable predictors of MACE and MACCE at one year.

Variables in SXSII model	MACE		MACCE	
	HR [95% CI]	P value	HR [95% CI]	P value
SXSII	1.055 [1.03-1.08]	<0.001	1.065 [1.04-1.094]	<0.001
MVD	1.837 [1.04-3.25]	0.037	1.714 [0.853-3.441]	0.130
Fasting Glucose	1.067 [1.01-1.13]	0.032	1.055 [0.99-1.19]	0.075
Resuscitation	3.571 [1.67-7.64]	<0.001	5.07 [2.41-10.65]	<0.001
Gender (Female)	0.329 [0.14-0.80]	0.012	0.314 [127-0.773]	0.012

H&L Test: X^2 :7,df:8,p:0.45(MACE), X^2 :4.067,df:8,p:0.85(MACCE)

Variables in SXS Model	MACE		MACCE	
	HR [95% CI]	P value	HR [95% CI]	P value
Age	1.064 [1.03-1.11]	<0.0001	1.058 [1.03-1.08]	<0.001
GFR	1.011 [1.01-1.02]	0.011	1.007 [0.997-1.016]	0.178
LVEF	0.956 [0.93-0.98]	<0.001	0.962 [0.94-0.98]	0.004
MVD	2.432 [1.37-4.29]	0.002	2.325 [1.18-4.58]	0.015
Resuscitation	5.55 [2.43-12.68]	<0.0001	8.12 [3.64-18.10]	<0.001
Fasting Glucose	1.07 [1.01-1.15]	0.022	1.06 [0.99-1.13]	0.059
SXS	1.017 [0.982-1.053]	0.343	1.006 [0.972-1.040]	0.746

H&L Test: X^2 :4.181,df:8,p:0.8(MACE), X^2 :3.91,df:8,p:0.865(MACCE)

Supplementary Table 3. GRACE versus SxSII score in prediction of 1-year All-cause mortality

Variables	All-Cause Mortality	
	HR [95% CI]	P value
GRACE Score (per point increment)	1.010 [0.98-1.03]	0.379
SxSII (per point increment)	1.061 [1.01-1.11]	0.014

Variables	All-Cause Mortality	
	HR [95% CI]	P value
GRACE score (per point increment)	1.00 [0.97 - 1.022]	0.615
$SxSII_{High}$ (SXSII ≥30.6)	16.3 [1.90 - 140.3]	0.01
